# Laser Deposition of Metal Oxide Structures for Gas Sensor Applications

**DOI:** 10.3390/ma19010176

**Published:** 2026-01-03

**Authors:** Nikolay Nedyalkov, Anna Dikovska, Tina Dilova, Genoveva Atanasova, Reni Andreeva, Georgi Avdeev

**Affiliations:** 1 Institute of Electronics, Bulgarian Academy of Sciences, 72 Tsarigradsko Shosse Blvd, 1784 Sofia, Bulgaria; 2 Institute of General and Inorganic Chemistry, Bulgarian Academy of Sciences, Acad. G. Bonchev Street, Bl. 11, 1113 Sofia, Bulgaria; tina@svr.igic.bas.bg (T.D.);; 3 Rostislaw Kaischew Institute of Physical Chemistry, Bulgarian Academy of Sciences, Acad. G. Bonchev Street, Bl. 11, 1113 Sofia, Bulgaria; randreeva@ipc.bas.bg (R.A.); g_avdeev@ipc.bas.bg (G.A.)

**Keywords:** laser-induced reverse transfer, deposition of oxide nanostructures, resistive gas sensors

## Abstract

This work presents results on laser-induced fabrication of metal and oxide structures on glass substrates. The Laser-Induced Reverse Transfer (LIRT) technique is applied using Zn and Sn, sintered ZnO and SnO_2_, and oxide composite targets. The processing is performed by nanosecond pulses of a Nd:YAG laser system operated at wavelength of 1064 nm. Detailed analyses of the deposited material morphology, composition and structure are presented, as the role of the processing conditions is revealed. It is found that at the applied conditions of using up to five laser pulses, the deposited material is composed of a nanostructured film covered in microsized nanoparticle clusters or droplets. The use of metal targets leads to formation of structures composed of metal and oxide phases. The adhesion test shows that part of the deposited material is stably adhered to the substrate surface. It is demonstrated that the deposited materials can be used as resistive gas sensors with sensitivity to NH_3_, CO, ethanol, acetone and N_2_O, at concentrations of 30 ppm. The ability of the method to deposit composite structures that consist of a mixture of both investigated oxides is also demonstrated.

## 1. Introduction

The common trend of miniaturization of modern electronic, optical and mechanical devices in recent years requires efficient technologies for fabrication of micro- and nanostructures of a large variety of materials [1,2,3]. The developed “top-down” and “bottom-up” approaches nowadays [4,5,6] may offer a spatial resolution down to the nanometer level, but generally, these technologies are still expensive, including complex tools, multiple steps and/or expressive precursors. The large variety of chemical methods (such as chemical vapor deposition, atomic layer deposition, sol–gel methods and other wet chemical approaches) that have advantages in productivity, reproducibility and structure control usually require multistep procedures, use of hazardous materials and expensive initial components and precursors. The physical methods based on lithography and gas-phase-based deposition techniques (particle-induced sputtering and thermal deposition) may offer high spatial resolution, large-scale production and good reproducibility, and based on these, are mainly considered in industrial microelectronics fabrication. These techniques also express some drawbacks related to consisting of multiple steps, complex sample preparation, expensive equipment and operation conditions (for fabrication and deposition, usually high vacuum). As an efficient alternative laser-based method for fabrication of micro- and nanostructures, they attract significant interest [7,8,9]. The present state of development of laser technologies defines an ability of precise processing of any known material [10]. The basic process in many laser technologies is ablation—a process in which laser radiation induces ejection of material from a target surface. This process can form the basis of both “top-down” and “bottom-up” approaches for structure formation. From one side, the ablated material could be composed of nanostructures such as nanoparticles and clusters that can be used in gas phase or in liquids. For more complex morphologies and structures, the ablated material could be collected on a substrate, as different processing parameters can be varied, resulting in the formation of desirable structures [11]. Furthermore, the ablation process can result in a structuring of the material that remains on the surface after ablation. In this “top-down” approach, complex nanostructures can be formed even in 3D [12]. A classic example of the former is pulsed laser deposition [13,14], where the laser irradiation ablates material from a target and it is deposited on a substrate, usually placed in front of the ablation plume. Modifications of this technique are termed laser-induced transfers, where the irradiated target and the substrate are placed a small distance apart, (from 0 to few tens of microns) [15,16,17,18,19,20,21,22,23,24,25,26,27,28]. In Laser-Induced Reverse Transfer (LIRT) [21,22,23,24,25], the laser radiation is focused on a target through a transparent substrate placed at a close distance. The ablated material is then deposited on the transparent substrate. Here, the main limitation is that the substrate should be transparent to the laser radiation, but no specific requirements for the target are imposed—it could be bulk material or thin film. The method is demonstrated for different laser sources with different wavelengths and pulse durations from fs to ns, as high-repletion-rate systems (kHz to MHz) are usually used. The main goals in these studies are related to fabrication of conductive areas on dielectric materials. LIRT is used for deposition of metals and more complex materials such as metal oxides—ITO [29], SnO_2_ [30], AlZnO [18] and brass [31]. It is also demonstrated that the method can be used for the formation of NiO_x_/TiO_y_ nanoparticles from a Ni/Ti alloy target [32]. The works reported show that even with a simple experimental scheme, the laser-induced transfer technique involves complex physical and chemical processes, including laser energy absorption mechanisms, intensity light modulation due to interference in the gap between the substrate and the target, ablated material dynamics in this complex geometry, deposition and redeposition effects, and the role of oxidation and other chemical reactions between the ablated material and the substrate or with the environment gas. Since the main goal is the formation of high-density deposits, that would ensure good electrical conductivity, the process at a low pulse number is rarely discussed. Based on the complex nature of processes involved in LIRT, the full capacity of the technique is still not fully revealed.

In this work, we study the ability of deposition of structures from Zn, Sn, ZnO, SnO_2_ and oxide composite targets by using LIRT. The materials are chosen with the aim of developing an easy fabrication of Zn and Sn oxides, materials that attract significant interest in the design and development of devices in photonics and electronics, and deposition on a cheap substrate as it is glass. As the process is realized in air, the work also aims to clarify whether the direct formation and deposition of oxides is possible using metal targets. The LIRT method is chosen as a simple experimental approach and is capable of fabrication of structures that are highly adhered to the substrate. This property would address the problem of stability of the deposits, generally needed for practical applications. Since these materials are rarely considered so far in LIRT (SnO_2_, except when a powder target and CW laser [30] are used, and Zn [24]), and also the ability for deposition of complex oxide systems is not addressed, the present work would shed new light on this fabrication method’s capabilities in general and on its ability for deposition of particular materials with a broad range of applications. Furthermore, the technique is applied for a small number of pulses, up to five, which has not been considered previously but may have a strong impact of the cost efficiency and speed of structure fabrication. In order to estimate the potential of the process and the deposited materials, an estimation of their application as resistive gas sensors is demonstrated.

## 2. Materials and Methods

**Figure 1:**
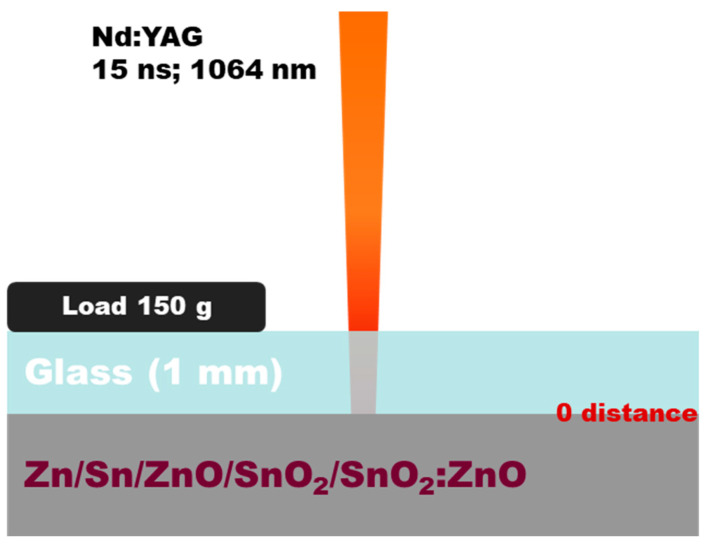
Configuration of the experimental setup for LIRT used in this study.

The experiments performed are based on a classical LIRT configuration (Figure 1). They follow two main directions: In the first, irradiation of metal targets of Zn and Sn, which are in the form of plates with a thickness of about 1 mm and polished with a surface roughness of Ra ≈ 0.5 µm, is performed. In the second, the target materials are sintered ceramics of ZnO and SnO_2_, and composite ones with compositions 10 wt%SnO_2_ in ZnO (referred to as 10SnZn in the text) and 10 wt%ZnO in SnO_2_ (referred to as 10ZnSn in the text). The targets are custom-prepared(homemade) from pure ZnO (Merck #1.08849, Merck, Darmstadt, Germany ) and SnO_2_ (Merck #1.07818, Merck, Darmstadt, Germany) powders, and their mixture in ratios 10 wt% SnO_2_ in ZnO and 10 wt% ZnO in SnO_2_. The powders are transformed into tablets by cold pressing at 100 MPa in air. All targets are calcified at 500 °C for 4 h and, subsequently, synthesized at a temperature of 1200 °C for ZnO-based targets and at 1000 °C for SnO_2_-based targets. The surface roughness of the prepared samples is estimated to about Ra ≈ 2 µm. The use of the composite target is considered in order to estimate the ability of the method for deposition of complex materials. The substrate is soda-lime microscope slide glass with a thickness of about 1 mm, which is placed directly on the target (i.e., the distance between the target and the substrate is approximately 0). In order to prevent motion of the substrate during laser processing due to induced shock waves, a load of about 150 g is placed on the top glass surface. The laser processing is performed by a Nd:YAG laser system (LotisTii LS-2147, Minsk, Belarus) that delivers laser pulses with a duration of 15 ns. The laser radiation is at the fundamental wavelength of 1064 nm, as the intensity distribution corresponds to multimode. It is directed through the glass and focused on the target by a lens with a focal distance of 30 cm. The target material is not placed in the focal plane, but the distance between the lens and the target is 20 cm, ensuring a spot with a size of about 2 mm on the target, measured by the induced spot on sensitive paper. This spot size results in a sufficient surface area of the deposited material that allows easy characterization.

The performed experiments are carried out by varying the laser parameters—the laser fluences and pulse number. Since in the presented studies so far the LIRT method for application of a small number of pulses has not been studied in detail, in this work this parameter is changed up to 5. The disposition is carried out in percussion mode, i.e., laser pulses are applied in a fixed position, and no scanning is applied.

The surface morphology of the deposited material is analyzed by scanning electron microscope (SEM) (FEI Quanta FEG 250 (Waltham, MA, USA)) and (Zeiss EVO 15, (Oberkochen, Germany)), equipped with an energy-dispersive X-ray (EDX) spectrometer (Oxford Instruments, Abingdon, UK). The phase and chemical composition of the material in the deposition zones are studied by X-ray diffraction (XRD) (Bruker D8 Advance diffractometer, Billerica, MA, USA, CuKα radiation), EDX, and X-ray photoelectron spectroscopy (XPS) (AXIS Supra electron spectrometer, Kratos Analytical Ltd. (Manchester, UK)) equipped with deconvolution software (ESCApeTM 1.2.0.1325 of Kratos Analytical Ltd. (Manchester, UK)), respectively. The equipment-integrated software and databases are used for phase identification. The surface roughness of the target materials is measured by 3D optical profilometer Zeta-20 (Zeta Instruments, San Jose, CA, USA). The transmission spectra of the deposited material are measured using a system that consists of white light source DH 2000 (215–2500 nm) and a spectrometer HR 4000 (200–1100 nm), both from Ocean Optics (Orlando, FL, USA).

**Figure 2:**
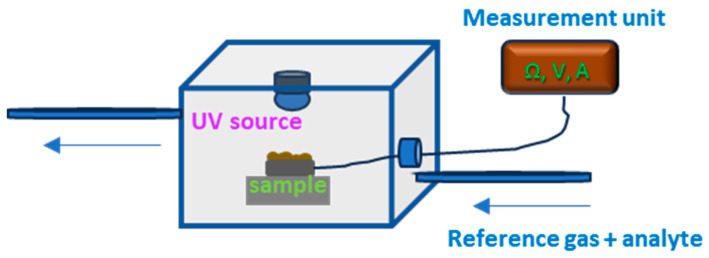
Scheme of the system used for resistive gas sensor properties of the deposited materials.

The gas sensing properties of the deposited materials are investigated by measuring the change in the resistance with the change in the gas environment in a homemade chamber. The optimal working conditions are obtained using irradiation by a 367 nm UV diode (intensity 200 mW/cm^2^) located at a distance of 1.5 cm from the sensor surface. A scheme of the gas sensor analysis system is presented in Figure 2. The measurements are performed with a Keithley 2450 SourceMeter (Keithley, Beaverton, OR, USA) and recorded in real time by a computer. Nitrous oxide (N_2_O), ammonia (NH_3_), carbon monoxide (CO), ethanol (C_2_H_6_O) and acetone ((CH_3_)_2_CO) with a concentration of 30 ppm are injected via a micro-syringe at room temperature in a flow of high-purity N_2_ reference gas. The gas response of the sensor element is determined as (ΔR/R0) × 100, where ΔR = Rg − R0, Rg is the sensor resistance in the test gas, and R0 is the sensor resistance in the reference gas environment after UV irradiation.

## 3. Results

### 3.1. Laser-Induced Reverse Transfer Using Zn and Sn Targets

#### 3.1.1. Surface Morphology

In the first type of experiments, the deposition is performed using Zn and Sn metal targets. It is found that there are processing value windows where efficient deposition can be performed. With respect to the laser fluence applied, a lowest value exists below which deposition is not realized. This value is related to the ablation threshold of the target material. For single-pulse processing, the estimated values are about 1.7 J/cm^2^ for Zn and 1.4 J/cm^2^ for Sn. The threshold value is defined as the fluence at which surface modifications on the target surface are observed under SEM. A set of experiments are conducted in a fluence range; for each fluence, three irradiations are performed. The threshold is defined as the minimal fluence at which surface modifications are observed in at least two of the spots. The increase in the laser fluence to values of about 15 J/cm^2^ results in morphology changes in the glass substrate in the zone of the target ablation. These values define the fluence processing window where experiments can be conducted.

**Figure 3:**
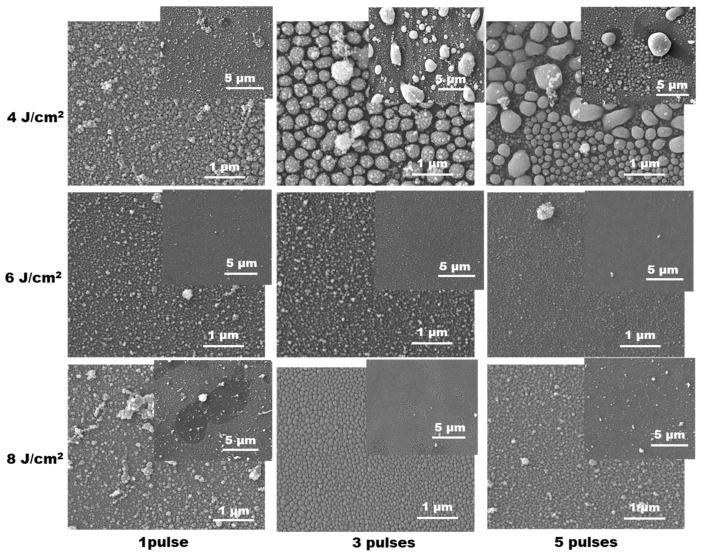
SEM images of the material deposited by LIRT of Zn at different processing conditions. For each case, images at two magnifications are presented.

Figure 3 represents SEM images of the glass surface in the deposition area for different processing conditions in the case of using a Zn target. The results present cases for three different laser fluences and application of one, three and five pulses. The images shown are for two magnifications in each case. The size distribution histograms for the structures presented in the images with the higher resolution are given in Figure S1 in the Supplementary Material. The SEM images reveal that the morphology of the deposited material depends on the processing conditions. At the lowest laser fluence of 4 J/cm^2^, the deposited material is composed of well-defined particles with a large variation in size. For single-pulse irradiation, they are mainly sub-micrometer in size and form a dense 3D cover. The largest structures observed are nanoparticle clusters. With the increase in the applied pulse number, microsized particles are observed, as the maximal size may exceed 5 µm. The surface of the substrate between these particles is covered by a finer component of well-defined spherical-like sub-micrometer particles with size in the range 100–500 nm in the case of application of three pulses. These particles are surrounded by smaller ones with an estimated diameter of a few tens of nanometers.

The application of five pulses at this laser fluence results in a large variation in the finer particles’ size, as it is in the range from about 1 µm to tens of nanometers. Based on the above, it can be concluded that in the case of using deposition at 4 J/cm^2^, the increase in the applied pulse number leads to an increase in the maximal size and the size distribution of the finer component. For the other two fluences presented in Figure 3, a general characteristic is that deposition of microsized particles is negligible. Instead, the substrate surface is homogeneously covered by a dense layer of nanoparticles with sizes in the range up to about 100 nm. Nanoparticle clusters with larger size can also be seen.

**Figure 4:**
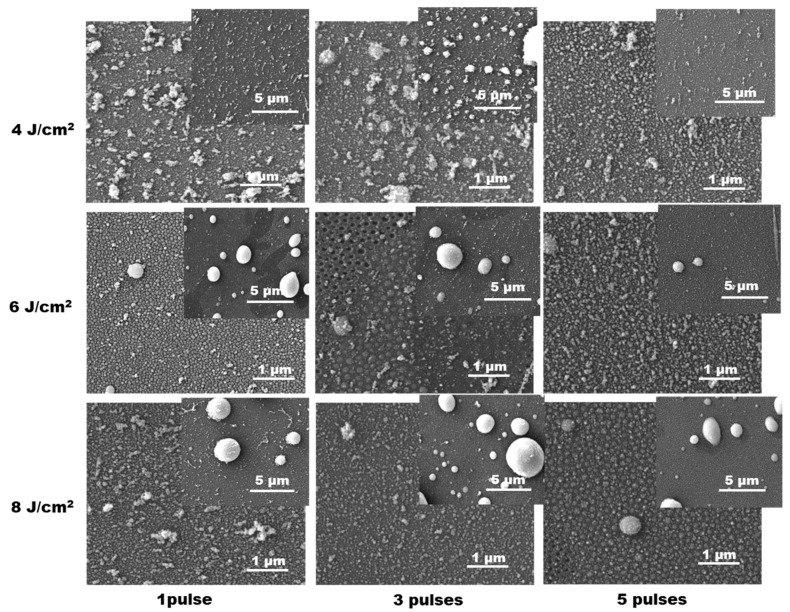
SEM images of the material deposited by LIRT of Sn at different processing conditions. For each case, images at two magnifications are presented.

In the case of LIRT of Sn, the deposited material can also be defined as composed of micro- and nanoparticles. Figure 4 shows SEM images of the deposited material at different processing conditions. Here, images at two magnifications for each experimental condition are also presented.

The size distribution histograms for the structures presented in the images with the higher resolution are given in Figure S2 in the Supplementary Material. As is seen in the SEM images, the deposition of Sn in most of the cases results in formation of microsized particles. At the lowest fluence of 4 J/cm^2^, these are clusters composed of smaller particles or homogeneous spherical-like droplets. With the increase in the laser fluence, the presence of the latter becomes clearer as the number density of such structures and their size increase. The finer component, which is also presented here as in the case of Zn deposition, is also characterized by sub-micrometer size. It should be mentioned that in some cases presented in Figure 4 (laser fluence of 6 J/cm^2^ and application of three laser pulses, and laser fluence of 8 J/cm^2^ and application of five laser pulses), the nanoparticles seem embedded in the substrate. In some places, the formation of nanoholes is clearly observed. Since their size corresponds to neighboring embedded nanoparticles, it can be concluded that these holes are formed by ejection of these nanoparticles.

#### 3.1.2. Composition and Structure of the Deposited Material

**Figure 5:**
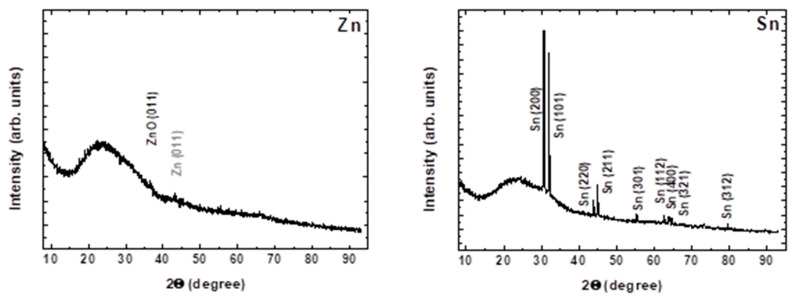
XRD spectra of the material deposited in the LIRT experiment with Zn and Sn targets. The process is realized at a laser fluence of 4 J/cm^2^ and with application of 5 laser pulses.

The composition and the structure of the deposited material are revealed by performing XDR and XPS analyses. Figure 5 shows XRD spectra for deposition from Zn and Sn targets, respectively, at a laser fluence of 4 J/cm^2^ and with application of five pulses. In both cases, peaks of the metal phase are defined. In the case of Zn, the peak intensity is low, probably due to the lower amount of deposited material. Here however, a better-defined peak of the oxide phase, ZnO (011), is observed.

**Figure 6:**
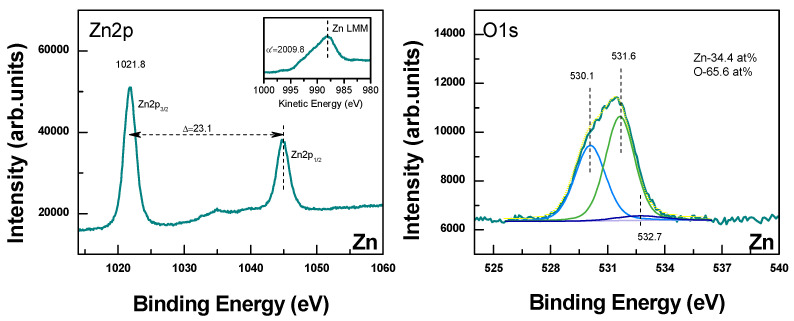

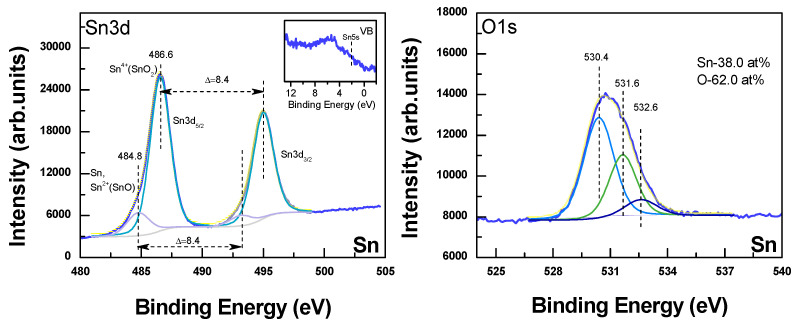
Zn2p, Sn3d and corresponding O1s XPS spectra of the material deposited by LIRT at a laser fluence of 4 J/cm^2^ and with application of 5 laser pulses from Zn and Sn targets, respectively. In the graphs related to Zn, the green spectrum is the as measured, yellow is the fitted, and blue, light green and dark blue are the deconvolution components. In the case of Sn spectra, the blue spectrum is the as measured, yellow is the fitted, and the green, grey, light and the dark blue correspond to the components from deconvolution.

Further clarification of the deposited material composition is given by the performed XPS analysis. Figure 6 represents Zn2p and O1s core level for the structure deposited from the Zn target, and Sn3d and O1s when Sn is used. The peaks in the Zn2p spectrum that correspond to Zn2p3/2 and Zn2p1/2 are located at 1021.8 eV and 1044.9 eV, with spin-orbit splitting of 23.1 eV. These parameters indicate that the Zn atoms are in the Zn^2+^ oxidation state [33,34]. For definition of the chemical state of Zn the Modified Auger Parameter α′ = (binding energy of Zn 2p3/2) + (kinetic energy of Zn L3M45M45) is used. The obtained experimental value of 2009.8 ± 0.1 corresponds to Zn^2+^ (ZnO) [35]. The peak in O1s spectra at 530.1 eV can be attributed to the Zn–O bonding. The high-energy peaks located at 531.6 eV and 532.7 eV could be attributed to the OH group and water absorbed onto the surface of ZnO [36]. Using the integrated equipment software, the composition of the surface structure is estimated as about 34% Zn and 66% O.

In the case of using a Sn target, the two peaks in the Sn3d spectrum with positions 486.6 eV and 495.2 eV can be identified as Sn 3d_5/2_ and Sn 3d_3/2_, respectively. The spin-orbital split is 8.4 eV. These data lead to the conclusion that the valence state is Sn^4+^ [37]. A low-intensity peak at 484.8 eV can also be identified. This shows that an additional state of Sn, such as Sn^2+^, and the respective formation of SnO, could be presented at the surface of the deposited material. An indication of the existence of Sn^2+^ can be seen from the slope of the valence band spectra (insert in Sn3d spectra). According to Ref. [38], SnO is characterized by a peak in Sn5s at about 2 eV. It should also be mentioned that due to peak overlapping, presence of metal Sn could also not be excluded.

The results from XRD and XPS analyses indicate that deposited material obtained when metal targets are used consists of metal phase and oxide. The latter is probably located mainly on the surface, since the XPS analysis gives information from the top few atomic layers of the material [39], while for XRD, the penetration depth is in the micrometer-scale range [40].

**Figure 7:**
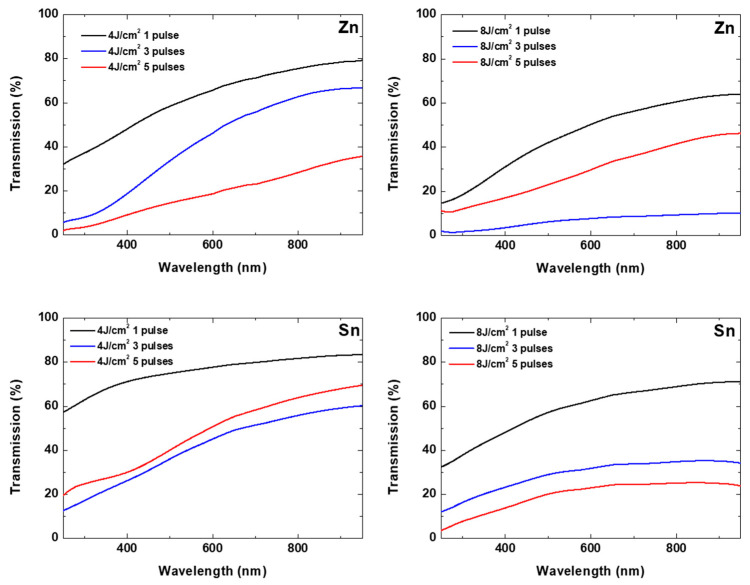
Transmission spectra of structures deposited under different processing conditions. The target material and the experimental parameters are presented.

Further information about characteristics of the deposited material can be extracted from their transmission spectra. Figure 7 shows the transmission spectra of structures deposited from Zn and Sn under different experimental conditions. The SEM images of these structures are given in Figure 3 and Figure 4, respectively. As the presented analyses suggest, the deposited materials are composed of a metal core and metal oxide on the structure’s outer surface. The presented transmission spectra indicate a decreased transmission with the decrease in the wavelength in the presented spectral range, which is typical for non-noble metallic thin films. No clear abrupt decrease in the transmission in UV that could correspond to a band gap of the related oxides is observed. It can be considered that the transmission is inversely proportional to the amount of the material in the investigated area. In the case of Zn ablation, at the laser fluence of 4 J/cm^2^, the transmission decreases in all ranges with the increase in the applied pulse number. It can be concluded that the amount of the deposited material is higher for larger pulse numbers. This is not the case when the laser fluence is 8 J/cm^2^, where the transmission after application of three pulses is lower compared to the case of application of five pulses. This could be explained with ablation of deposited material during the processing. In the case of Sn, such an effect is observed for a laser fluence of 4 J/cm^2^, while at 8 J/cm^2^, the transmission of the deposit decreases with the pulse number increase. These results indicate that the process of formation of the structures in LIRT is realized by deposition on the substrate, but also ablation of the deposited material.

### 3.2. Laser-Induced Reverse Transfer Using Oxide Targets

#### 3.2.1. Surface Morphology

**Figure 8:**
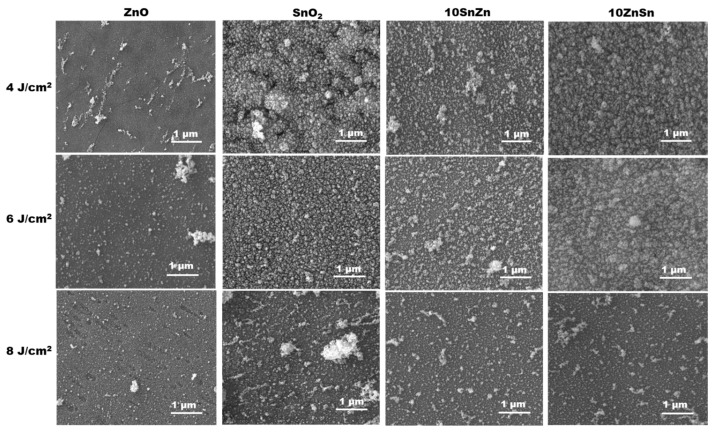
SEM images of the material deposited by LIRT of oxide targets under different processing conditions. The experiments are performed using 5 laser pulses. The target and the used laser fluences are indicated.

In the case of ablation from oxide targets, similar procedures of experiments and analyses are executed. It is found that the ablation thresholds for single-pulse processing are similar for the four synthesized targets—about 1.6 J/cm^2^. Even the absorption of the oxides is smaller compared to metals at the used wavelength; the ceramic structure of the target results in multiple scattering, which leads to more efficient absorption in the surface layer. Figure 8 represents SEM images of the deposited material for the different targets, for different laser fluences and a fixed pulse number of five. The size distribution histograms for the structures presented in the images are given in Figure S3 in the Supplementary Material. The performed experiments indicate that at the presented conditions LIRT is not accompanied by deposition of microsized droplets as in the case of metal targets (Figure 3 and Figure 4). In all cases presented in Figure 8, the deposited material is composed of a dense cover of nanoparticles, which is further covered in nanoparticle clusters with sizes that reach about a micrometer. In the case of using SnO_2_ and 10ZnSn targets and fluences of 4 J/cm^2^ and 6 J/cm^2^, the dense nanoparticle cover is 3D.

#### 3.2.2. Composition and Structure of the Deposited Material

**Figure 9:**
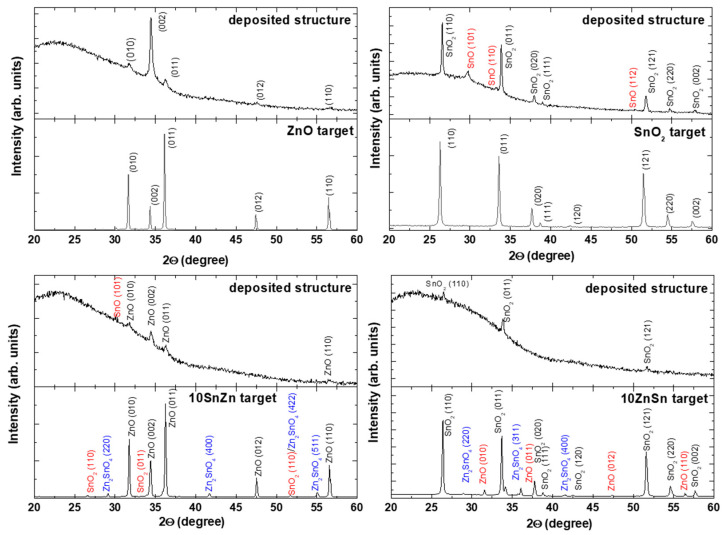
XRD spectra of the material deposited in the LIRT experiment with different oxide targets at a laser fluence of 4 J/cm^2^ and with application of 5 laser pulses. The spectra of the targets are also shown.

**Figure 10:**
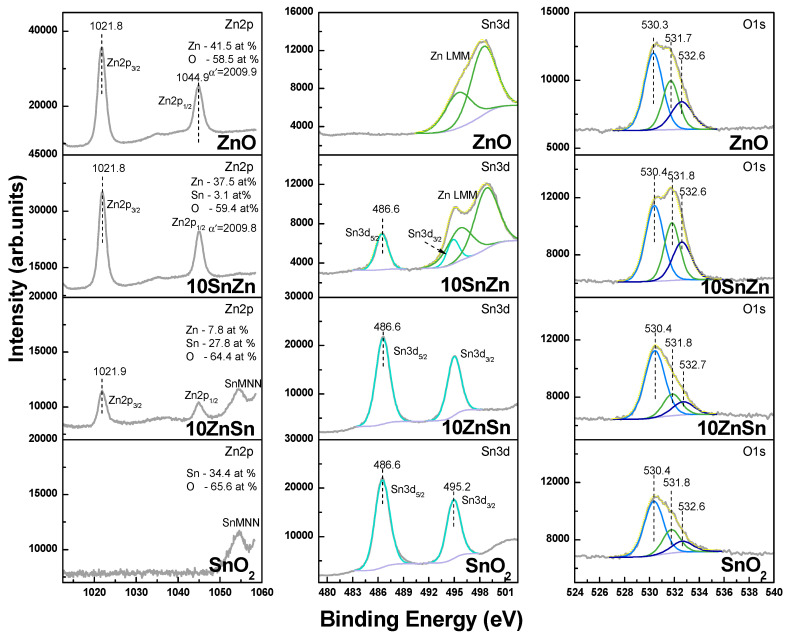
Zn2p, Sn3d and corresponding O1s XPS spectra for materials deposited from oxide targets by LIRT at a laser fluence of 4 J/cm^2^ and with application of 5 laser pulses. The target type is presented in each figure. The grey lines represent the measured spectrum, yellow is the fitted, and the green, light and dark blue correspond to the components of deconvolution.

The XRD spectra of the deposited materials when oxide targets are used are given in Figure 9. The experiments are performed at a fluence of 4 J/cm^2^ and with application of five laser pulses. The spectra of the fabricated targets are also presented. In the case of using the ZnO target, the deposited material shows a predominant orientation (200). The results for the other targets indicate that the deposition under the presented conditions results in a non-stochiometric material transfer. The SnO_2_ ablation results in the formation of SnO in the deposited material. For mixed oxides, an additional phase of Zn_2_SnO_4_ is identified in the target material. It is, however, not detected in the deposited material. When LIRT is performed with the 10SnZn target, the material on the substrate is composed of ZnO and SnO. In the case of the 10ZnSn target, the ZnO phase is not identified. It should be mentioned that the thickness of the deposited material in the presented experiments is in the order of a few tens to a few hundred nanometers, estimated by the size of the nanoparticles that form the structures. The low amount of the deposited material results in low intensity of the XRD spectra peaks and could make the detection of the low-concentration components difficult. In order to check more precisely the composition of the deposited material, XPS spectra are also measured. Figure 10 represents Zn2p, Sn3d and O1s XPS spectra for materials deposited from ZnO, 10SnZn, 10ZnSn and SnO_2_ targets, each at a laser fluence of 4 J/cm^2^ and with application of five laser pulses. Each row in Figure 10 shows the data for the specific target. In the case of composite targets, peak fitting of the spectra is carried out since the Sn3d and ZnLMM Auger peaks overlap. For the ZnO and SnO_2_ cases, the ZnLMM and SnMNN Auger electron spectra are also shown. Based on an analysis similar to that performed for Figure 6, it can be concluded that the spectral characteristics obtained indicate formation of only oxides in the deposited materials in all cases. When composite targets are used, the presence of both oxides is detected. The XPS analysis shows that on the surface of the deposited structures when targets of SnO_2_ and composite are used, SnO is not detected.

**Figure 11:**
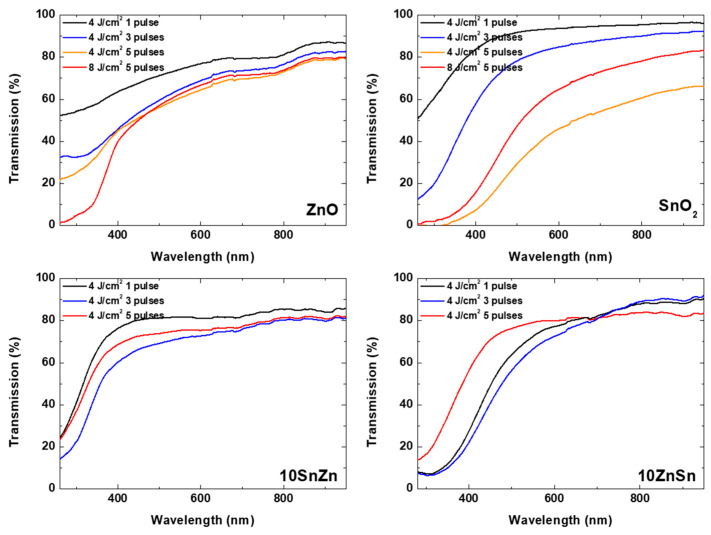
Transmission spectra of structures deposited at different processing conditions for different oxide targets. The target material and the experimental parameters are presented for each case.

The optical transmission spectra of the deposited structures using different oxide targets are presented in Figure 11. Results for different processing conditions are shown. Unlike the case of metal ablation, here a sharp decrease in the transmission in UV spectral range is observed. This is related to the band gap in the oxide materials used. The transmission values in this range could be related to the thickness of the deposited material. A detailed inspection of the dependence of the transmission spectra on the processing conditions indicates that increasing the pulse energy (in the case of SnO_2_) or pulse number (in the cases of 10 SnZn and 10 ZnSn) does not result in a decrease in the transmission value (increase in the deposited material thickness). As in the case of ablation of metal targets, this effect is related to ablation of the deposited material by consecutive pulses. Even the oxide particles deposited on the surface of the substrate have low absorption at the used laser wavelength; the effect of their ejection seems efficient, probably due to a lower adhesion, and enhanced absorption due to the formation of a porous morphology as is seen in Figure 8 for SnO_2_ and 10 ZnSn.

The ablation at presented conditions leads to formation of nanostructured film on the surface of the glass substrate for both types of targets—metals and metal oxides. In the case of ablation of metals Zn and Sn, formation of large amount of microsized droplets is also realized. These are not present in the case of ablation of oxide targets. The result can be explained by the low melting temperature of the used metals, 419.5 °C and 231.9 °C for Zn and Sn, respectively. During the ablation process, a significant part of the irradiated zone and the surrounded area consists of melt. The induced high pressure during ablation results in melt ejection of liquid droplets that solidify on the substrate as microsized spherical particles. The lower melting temperature of Sn can explain the larger microparticle density compared to Zn, as is seen in Figure 3 and Figure 4. In the case of oxide targets, the melting and the boiling points do not differ significantly as in the case of metals. Furthermore, the heat conduction of the oxide sintered targets is more than one order of magnitude lower than the metals [41]. This results in a reduction in the amount of melt when oxide targets are considered. The mechanism of formation of the nanoparticles observed in all cases studied is more complex. The experiments on laser ablation in air at similar conditions [42] indicate that formation of nanoparticles and clusters should be expected in the ablation plume due to condensation and interaction of the ejected material. Evidence for realization of nanoparticle interaction here is the formation of irregular nanoparticle clusters that can be seen in all SEM images presented. The sizes of nanoparticles are in the range of a few tens of nanometers and are quite similar for the different materials used. However, in some cases of ablation of metal targets, the dense cover consists of larger nanoparticles that form a well-expressed monolayer (for example, Figure 3, three pulses at fluences of 4 and 8 J/cm^2^; Figure 4 laser fluence of 6 J/cm^2^—one and three pulses). It can be proposed that these structures are formed by annealing of the thin metal film deposited by previous pulses. The method of laser dewetting of thin metal films [43] is demonstrated to lead to the formation of a monolayer of nanoparticles with a size that is defined by the film thickness. The basic physics underlaying the process is the poor wetting of the liquid metal to the oxide substrate. When the film is heated by the laser radiation, it is rapidly decomposed into nanoparticles. It is interesting to note that the dense nanoparticle layer is stably attached to the substrate, as in some cases it is obvious from SEM images that the particles are partially embedded in the glass surface (Figure 4, 6 J/cm^2^—three pulses; 8 J/cm^2^—five pulses). Since such effect is not observed after single-pulse irradiation, it can be assumed that its origin includes interaction of the deposited material with the next laser pulses. When the first laser pulse induces ablation at zero target-to-substrate distance, the ejected material is just transferred on the substrate; since there is no space for expansion, it presents a dense layer. Well above the ablation threshold, as is the case here, and for the present pulse duration, the ablation process starts before the end of the laser pulse [25]. The material deposited on the substrate interacts with the remaining part of the pulse, which may lead to decomposition into nanoparticles as in the dewetting process for metal films or morphology-induced changes by the heating of thin oxide films [44]. The latter are related to partial or complete melting and followed by recrystallization that is accompanied by expansion and contraction. These dynamics could result in transformation of the film into a structure of well-defined nanoparticles. These processes lead to formation of a discrete structure on the substrate. As the target material and the substrate are in contact, the glass substrate is also efficiently heated when the target absorbs laser irradiation. This results in a softening of the glass surface layer, which is achieved at temperature of about 1400 °C. The heating dynamics of the system considered here are studied by a numerical model based on a heat conduction equation [25] (see Supplementary Material, Figure S4). For the case of Zn, using thermophysical and optical parameters given in [45,46,47], the maximal achieved temperature at the target surface reaches the glass-softening temperature range at laser fluences of about 2.5 J/cm^2^. The contact between the hot target surface and the substrate, as is used in the experimental configuration, ensures an additional way of heating the deposited material which accelerates the film disintegration. Once such a structure is formed, it can be embedded into the glass surface due to the high pressure induced by the next laser pulses and softening of the glass. The interaction between the deposited material and the consecutive laser pulses can be validated by the change in the transmission of the deposited structures as shown in Figure 7 and Figure 11. These results also indicate that the process is self-limiting, i.e., when the thickness of the deposited material increases, its absorption also increases, which under certain conditions would result in ablation of part of the deposited film. It should also be mentioned that the ablation process, even after a single pulse, induces formation of a hole or irregularities on the target surface, which induces enhanced target-to-substrate distance. In this gap, the ablated species from the next laser pulse could lead to ablation material condensation into nanoparticles and formation of the observed nanoparticle clusters.

**Figure 12:**
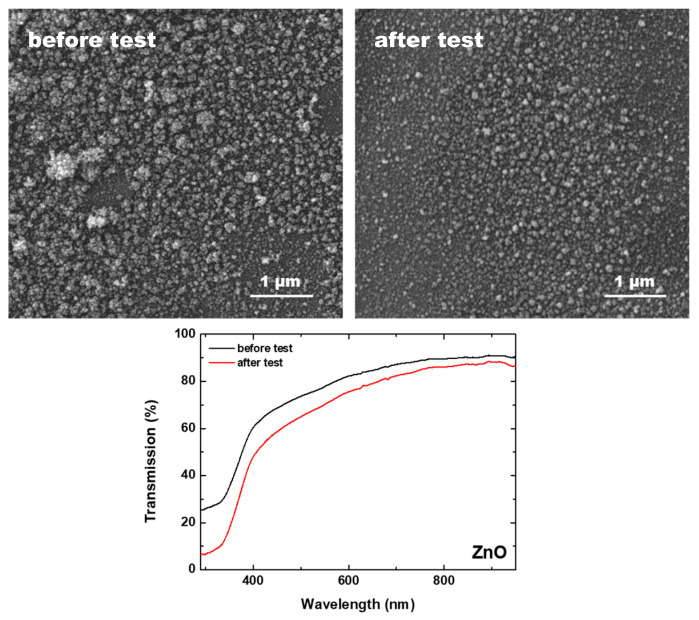
SEM images of the structure deposited from the ZnO target at 8 J/cm^2^ and with application of three laser pulses, before and after a Scotch tape test. Transmission spectra for both cases are also shown.

It is found that the dense film of nanoparticles that covers the substrate in all cases presented in this work when the pulse number is three and higher expresses high adhesion. To quantify this, a Scotch tape test is performed, in which Scotch tape (3M Scotch Adhesive Tape 200) is pressed on the deposited material and then removed. Figure 12 represents the SEM images and the transmission spectra before and after the Scotch tape test. The deposition is performed with a ZnO target at a fluence of 8 J/cm^2^ and with application of three pulses. The test indicates that part of the deposited material is removed, but part remains. Consecutive tests on this structure show that the structure is stable, and significant changes in the transmission spectrum are not further observed. The size distribution histograms for the structures presented in the SEM images in Figure 12 are given in Figure S4 in the Supplementary Material. These represent the distribution of the finest particles with sizes of tens of nanometers. The observed bigger clusters are composed of nanoparticles with the same dimensions. In the Scotch tape test, the size distribution of the finest particles remains (Figure S4), and particle clusters are removed, as is clearly seen in Figure 12.

The analyses of the composition of the deposited material indicate that under LIRT in air, the deposition of metal results in formation of a metal nanostructure which is oxidized only on the surface. Zuo et al. [30] demonstrated that LIRT can be used for the formation of Ni/Ti oxide nanoparticles by ablation of a metal alloy in their case. The authors explain that the oxidation occurs during the expansion of the ablated species in the gap between the target and substrate. In the experiments performed here, such oxidation is not realized in the whole nanoparticle volume. This is an indication that the LIRT method can be used for formation of core–shell structures with characteristics defined by the experimental conditions.

### 3.3. Resistive Gas Sensor Properties

In order to demonstrate the potential of the fabricated structures in real application, their efficiency as resistive gas sensors is investigated. The produced structures are placed in a chamber (see Figure 2), illuminated by UV light, and their resistance is measured after consecutive injection of different gases. The initial resistance, R0, is in the range from kΩ to MΩ, depending on the sample. The role of the UV light in the sensor performance is to generate electron–hole pairs in the oxides and to enhance desorption of the surface oxygen species [48]. The first is observed experimentally by a reduction in the samples’ resistance after illumination. For example, the illumination of the samples deposited from ZnO and SnO_2_ targets at a fluence of 4 J/cm^2^ and with application of five pulses with UV light results in a reduction in the resistance of about 1.3 and 2 times, respectively.

**Figure 13:**
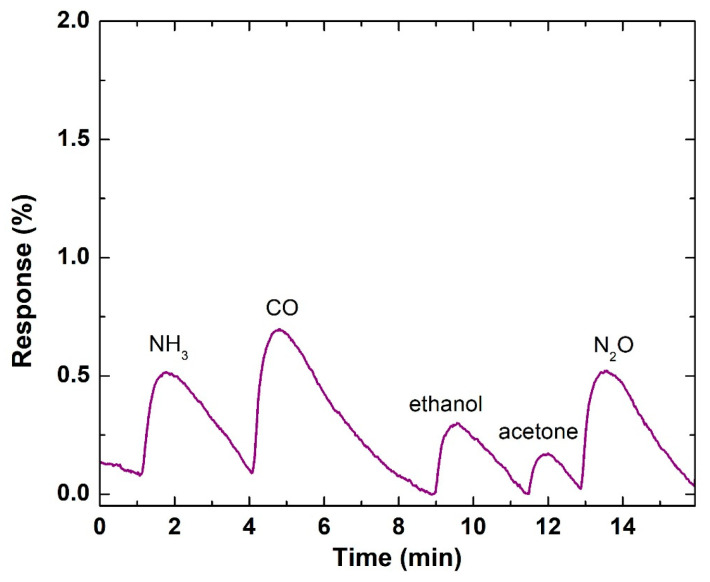
Response of a structure deposited from the Zn target at a laser fluence of 4 J/cm^2^ and with application of five pulses, for different gases, all at a concentration of 30 ppm. During measurement, the sample is under UV irradiation at a wavelength of 367 nm.

When deposition from a metal target is considered among the as-deposited structures, only in the case of deposition from the Zn target at a laser fluence of 4 J/cm^2^ and with application of five pulses are gas sensor properties observed. Figure 13 represents the response of this element to different gases each at concentration of 30 ppm. The results indicate that the deposited material expresses sensor properties for all used gases.

**Figure 14:**
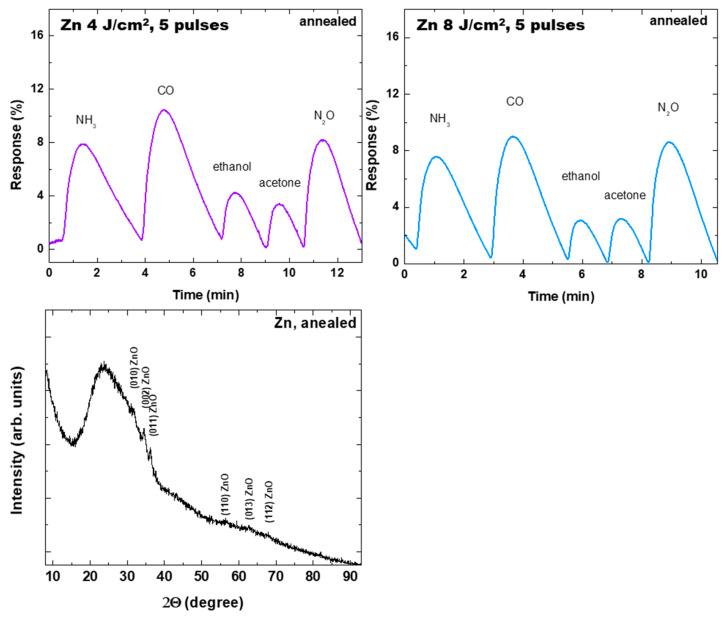
Response of structures deposited from the Zn target under different conditions for different gases, all at concentration of 30 ppm. The samples are annealed at 300 °C for 10 min in air. The processing conditions for fabrication are given. The XRD spectrum of the sample fabricated at a laser fluence of 4 J/cm^2^ and with application of 5 pulses and annealing is also shown.

A significant improvement in the sensor properties is obtained after annealing of the sample. This is conducted at 300 °C for 10 min, in air, for structures deposited from the Zn target. The change is related to an enhancement of the response of the sample presented in Figure 13, but also to expression of a clear sensor behavior for other structures that do not show it before annealing. This can be seen in Figure 14, where the response of different structures is presented. Note the difference in the response of the sample obtained from the Zn target at a laser fluence of 4 J/cm^2^ and with application of five pulses after annealing (compare with Figure 13) and the fact that the other structure does not show gas sensor properties before annealing. The increase in the response after annealing of the sample obtained at a laser fluence of 4 J/cm^2^ and with application of five pulses is more than an order of magnitude. The reason for the effect is the complete oxidation of the deposited structure, as can be seen from Figure 14, where the XRD spectrum of the annealed sample obtained at a laser fluence of 4 J/cm^2^ and with application of five pulses is also given. Furthermore, it is found that the annealing generally led to a decrease in the structure resistance, which could be attributed to a process of necking [49]. This may lead to better contact between nanostructures and to enhanced mobility of the carriers, which have a positive impact on the sensor performance.

**Figure 15:**
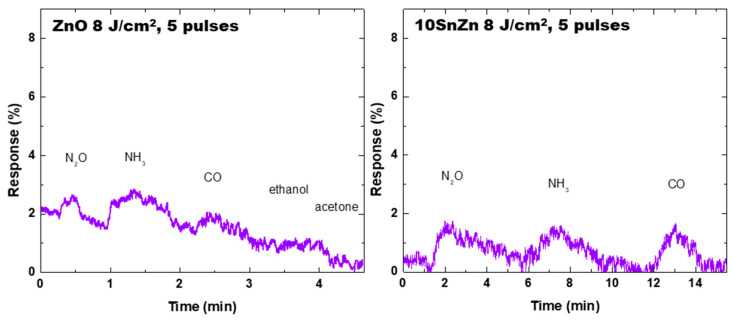
Response of structures deposited from ZnO and 10SnZn targets, for different gases, all at a concentration of 30 ppm. Before the gas sensor test, the samples undergo the Scotch tape test. The processing conditions for fabrication are given.

The results from gas sensor properties of the structures obtained when oxide targets are used are similar to the case presented in Figure 14. As it was demonstrated, the structures deposited express high adhesion after initial removal of debris by Scotch tape. In order to demonstrate the efficiency of the remaining structure in gas sensor application, the deposited materials are subjected to the Scotch tape test and then tested as sensors. Figure 15 shows results for two structures deposited from ZnO and 10SnZn targets. Even though the response is reduced after the Scotch tape test, the samples clearly demonstrate sensor ability. The reduction is related to a decrease in the amount of the sensitive material, but as is shown in Figure 12, part of it remains strongly attached to the surface. The sensor properties are also preserved after washing the structure with a water stream from a hand-pressed laboratory wash bottle. This is an indication that the structures formed by LIRT may have a higher service lifetime as sensors due to the ability to clean the sensitive element. Although the fabricated structures may be further optimized for better gas sensor performance, even the ones presented here can be used as a signal alarm in conditions when one of the presented gases is expected to occur. The structures presented in this work do not show clear selectivity for some of the used gases. This property could however be addressed by appropriate composition and/or morphology modifications [50,51].

Even though the fabrication method and the structure characteristics could be further optimized for the particular application as a gas sensor element, it worth comparing the performance of the obtained structures with those available in the literature. Table 1 shows values of some parameters of ZnO structures used as resistive gas sensors. Although the structure characteristics and the fabrication methods differ from those used in this work, some conclusions can be made. The element presented here has a good sensitivity expressed by the clear detection of the analyzed gases at a concentration of 30 ppm. The sensor also works at room temperature. As is shown, it can be cleaned, which is not offered by the other structures cited. These characteristics can be a basis for further interest in the presented structures and the method for the design and fabrication of sensor elements.

## 4. Conclusions

This work represents results on application of the method of Laser-Induced Reverse Transfer for deposition of nanostructured films from metal Zn and Sn, oxide ZnO and SnO_2_, and composite oxide 10 wt%SnO_2_ in ZnO and 10 wt%ZnO in SnO_2_ targets. The chosen geometry of touching target and substrate leads to formation of complex structures that express similarities for all targets used. The deposited material consists of a dense nanoparticle array which is stably attached to the substrate surface in all cases. As a second component, microsized droplets when metal targets are used, or nanoparticle clusters for the case of oxide targets, are formed. When Zn and Sn targets are ablated, the deposited material consists of metal and oxide phases, while for oxides only, oxide phases are observed in the deposits. The transfer is generally non-stochiometric as the presence of SnO is detected for targets initially composed of SnO_2_. The performed Scotch tape test leads to removal of part of the deposited material, mainly the second component composed of microsized droplets and nanoparticle clusters, but the dense nanoparticle film remains. Its presence is also not affected by water washing of the substrates. Some of the structures deposited from the Zn target express clear resistive gas sensor properties to different gases such as NH_3_, CO, ethanol, acetone and N_2_O. Their response is significantly enhanced when the structures are annealed at 300 °C for 10 min in air. It is also demonstrated that after the Scotch tape test, the remaining material can be used as a sensor element, which can respond to concentrations as low as 30 ppm. The demonstrated ability of deposition of oxides indicates that the method can be used for fabrication of more complex materials and, in this particular application, as a way of tuning the gas sensor performance of the structures by the composition.

## Figures and Tables

**Table 1:** Data about gas sensor parameters for a structure described in this work and some available in the literature.

Sample	Gas Type, Operation Temperature	Concentration (ppm)	Response (%)	Response Time (s)	Recovery Time (s)	Ref.
Zn, 4 J/cm^2^, 5 pulses, annealed	NH_3_	30	7.5	60	120	This work
	CO	30	9.8	36	126	
	Ethanol	30	3.4	20	56	
	Acetone	30	3.3	18	42	
	N_2_O, room temperature	30	8.1	24	78	
ZnO nanorods	CO, at ×100 °C	Above 50	-	-	-	[52]
ZnO nanoparticles	Ethanol, at 350 °C	400	20.3 (R0/Rg)	12	4	[53]
ZnO nanorods	Acetone, 172, 219 °C	100	12.9 (R0/Rg)	13	29	[54]
ZnO nanowires	NH_3_, room temperature	50	20 (R0/Rg)	88	65	[55]
ZnO nanoparticles	NH_3_, room temperature, UV irradiation	50	8 ((R0 − Rg)/R0) × 100	270	300	[56]
ZnO nanostructure	NH_3_	40	52	33–36	155–196	[57]
	CO	40	33			
	Ethanol	40	36			
	Acetone	40	54			
	Room temperature, UV radiation		((R0 − Rg)/R0) × 100			

## Data Availability

The original contributions presented in this study are included in the article/Supplementary Materials. Further inquiries can be directed to the corresponding author.

## Supplementary Materials

The following supporting information can be downloaded at: https://www.mdpi.com/article/10.3390/ma19010176/s1, Figure S1: Size distribution histograms for the structures presented in the high-resolution images in Figure 3; Figure S2: Size distribution histograms for the structures presented in the high-resolution images in Figure 4; Figure S3: Size distribution histograms for the structures presented in the high-resolution images in Figure 8; Figure S4: Size distribution histograms of the nanoparticles in the structure deposited from ZnO target at 8 J/cm^2^ and application of 3 laser pulses, before and after a scotch tape test. The SEM images are presented in Figure 12; Figure S5: Temperature evolution of the surface of Zn target that is in contact with glass substrate in LIRT experiment, for application of single laser pulse at different fluences.
