# Supplementary material for: Laser Deposition of Metal Oxide Structures for Gas Sensor Applications
Source: Materials (Basel). 2026 Jan 3;19(1):176. doi: 10.3390/ma19010176 (PMC12786783; doi:10.3390/ma19010176)
Supplement: Supplementary file 1 [file materials-19-00176-s001.zip › materials-4016617-supplementary.pdf]

Supplementary material

Laser Deposition of Metal Oxide Structures for Gas Sensor Applications, by Nikolay Nedyalkov et. al.

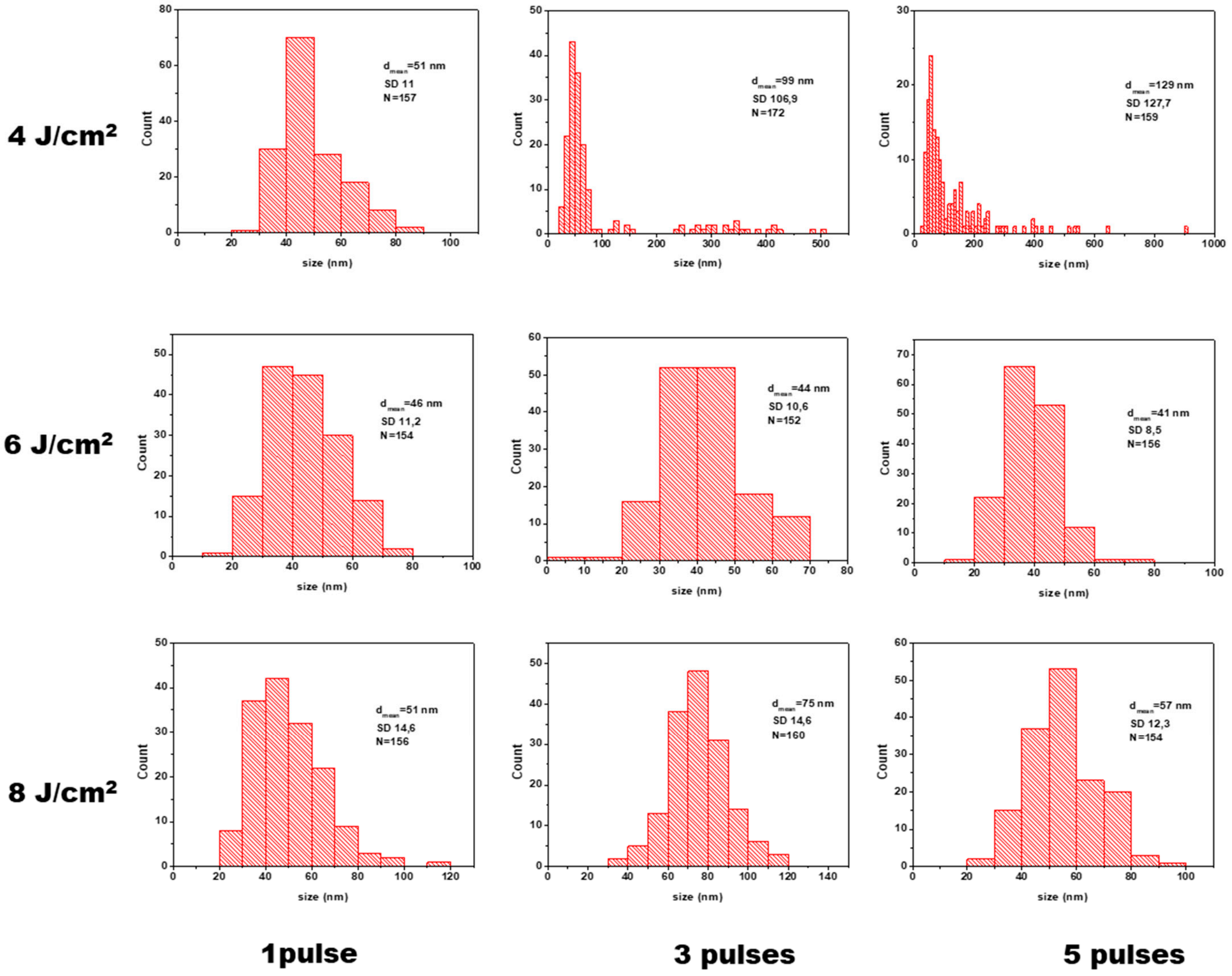

Fig. S1. Size distribution histograms for the structures presented in the high-resolution images in Fig. 3.

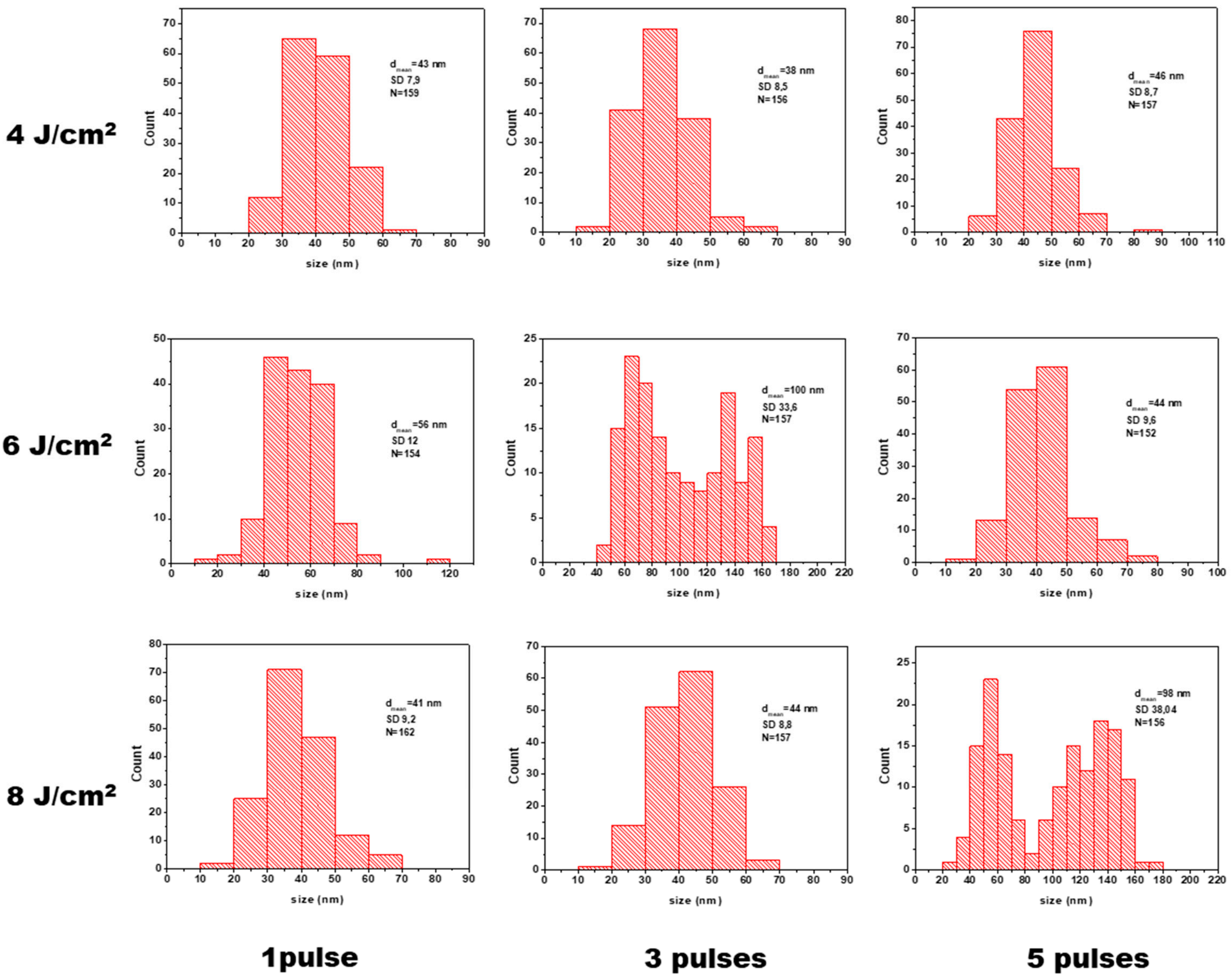

Fig. S2. Size distribution histograms for the structures presented in the high-resolution images in Fig. 4.

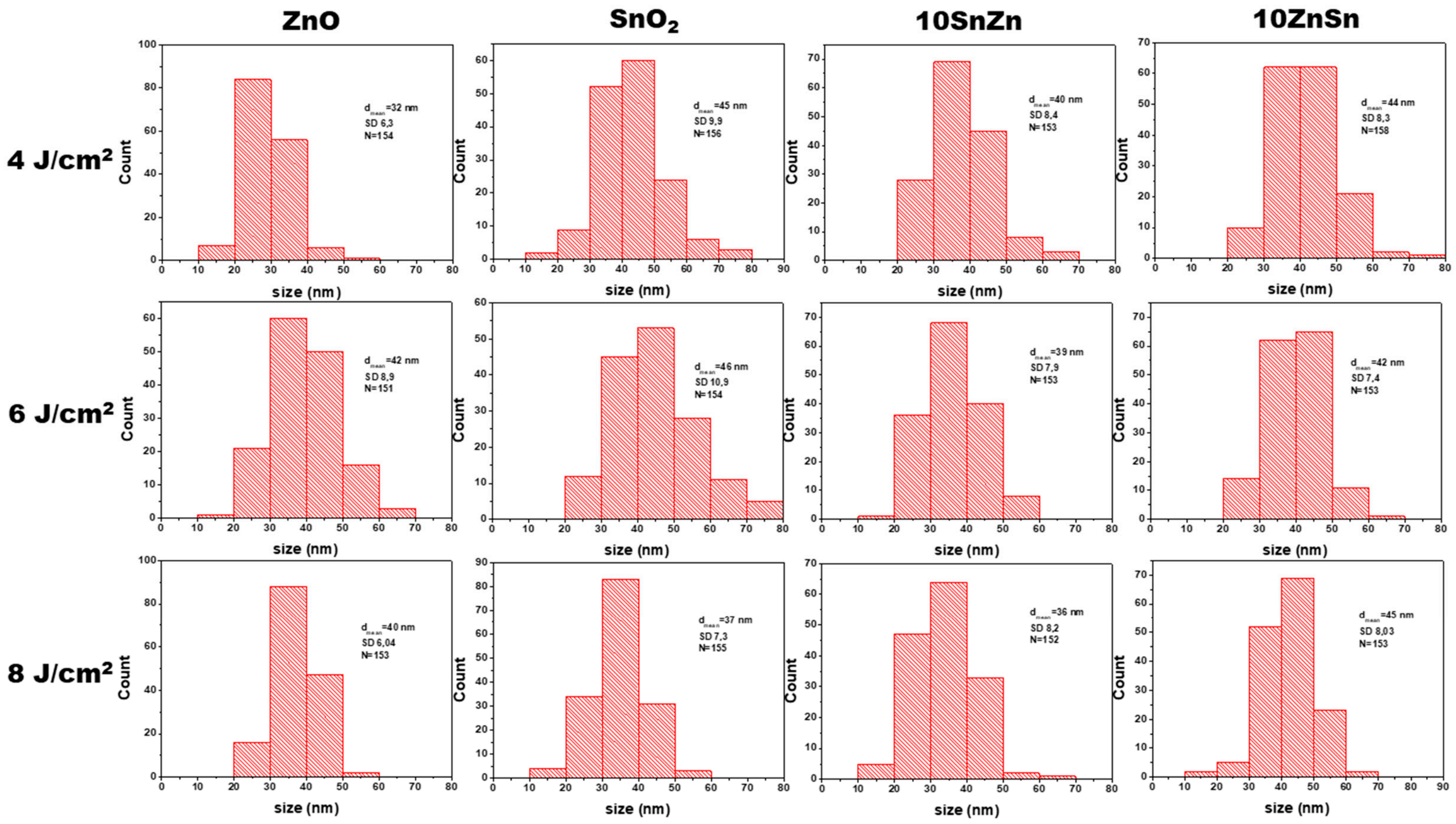

Fig. S3. Size distribution histograms for the structures presented in the high-resolution images in Fig. 8.

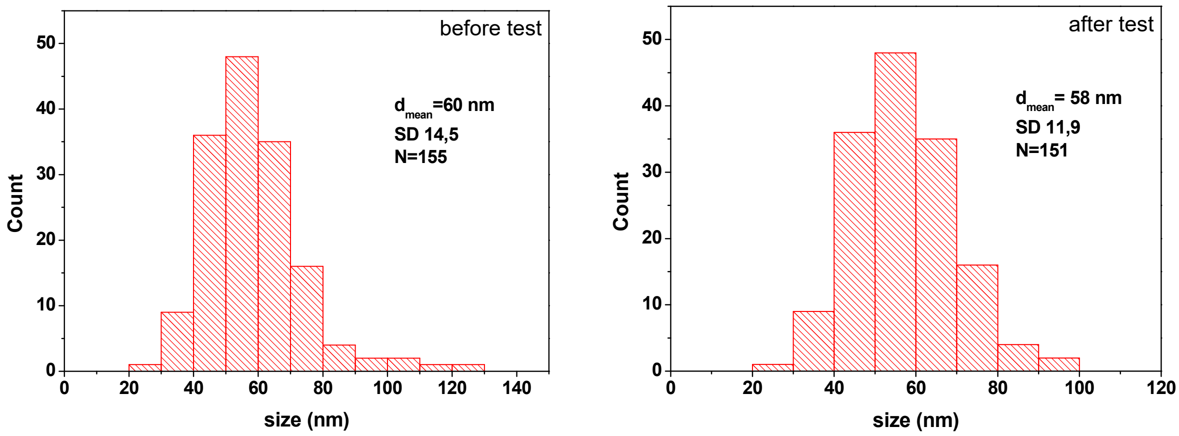

Figure S4. Size distribution histograms of the nanoparticles in the structure deposited from ZnO target at 8 J/cm^2 and application of 3 laser pulses, before and after a scotch tape test. The SEM images are presented in Fig. 12.

The temperature of the material at the contact between the target and the substrate gives important information about the formation mechanism and the reason for the good adhesion of the deposited material. In order to estimate this temperature a numerical model based on one dimensional heat conduction equation is applied [25]. Briefly, the model considers Zn metal plate and a glass one placed on it. The Zn and glass have thickness of 50 μm. The glass is considered to be transparent for the laser irradiation and only Zn absorbs. The laser pulse duration is 15 ns, the same as in the experiments. Figure S5 represents temperature evolution on the Zn surface after application of single laser pulse with fluence of 1.7 J/cm^2, which corresponds to the experimentally estimated ablation threshold, 2.5 and at 4 J/cm^2. The calculation results indicate that at the threshold fluence, the maximal temperature of the Zn surface approaches the boiling point (907^{0}C). This temperature is lower than the range where glass substrate softening and the ejected material is not embedded. This results in low adhesion and easy removal of the deposited material. The glass softening temperature range is reached at fluence of about 2. 5 J/cm^2. At the higher fluence, the temperature is sufficient for glass softening and the ablated material can be partially embedded into glass substrate.

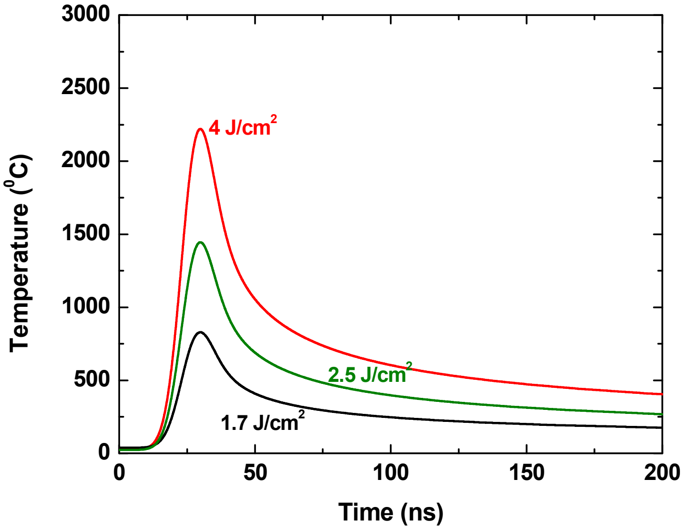

Figure S5. Temperature evolution of the surface of Zn target that is in contact with glass substrate in LIRT experiment, for application of single laser pulse at different fluences.
